# Decay Kinetics of Gd_3_Al_2_Ga_3_O_12_:Ce^3+^ Luminescence under Dense Laser Irradiation

**DOI:** 10.3390/ma16030971

**Published:** 2023-01-20

**Authors:** Dmitry Spassky, Andrey Vasil’ev, Nataliya Krutyak, Oleg Buzanov, Vladimir Morozov, Alexei Belik, Nikita Fedorov, Patrick Martin, Andrei Belsky

**Affiliations:** 1 Skobeltsyn Institute of Nuclear Physics, Lomonosov Moscow State University, Leninskiye Gory 1-2, 119991 Moscow, Russia; 2 Institute of Physics, University of Tartu, W. Ostwald Str. 1, 50411 Tartu, Estonia; 3 Physics Department, Lomonosov Moscow State University, Leninskiye Gory 1-2, 119991 Moscow, Russia; 4 Fomos-Materials, Buzheninova 16, 107023 Moscow, Russia; 5 Department of Chemistry, Lomonosov Moscow State University, Leninskiye Gory 1-3, 119991 Moscow, Russia; 6 International Center for Materials Nanoarchitectonics (WPI-MANA), National Institute for Materials Science, Namiki 1-1, Tsukuba 305-0044, Ibaraki, Japan; 7 Centre Lasers Intenses et Applications, Université de Bordeaux—Centre National de la Recherche Scientifique—Commissariat à l’Énergie Nucléaire, 33405 Talence, France

**Keywords:** GAGG:Ce, decay kinetics, Z-scan, excitons interaction, non-linear density effects

## Abstract

The decay kinetics of Gd_3_Al_2_Ga_3_O_12_:Ce^3+^ single crystal luminescence were studied under dense laser excitation. It was shown that the decay times as well as the intensity of Ce^3+^ luminescence depend on the excitation density. The observed effects were ascribed to the interaction between excitons as well as to the features of energy transfer from the excitons to Ce^3+^. The numerical simulation of the experimental results was performed for justification of the proposed model.

## 1. Introduction

Gd_3_Al_2_Ga_3_O_12_:Ce^3+^ (GAGG:Ce) scintillator is a high-density chemically stable compound with a high scintillation yield (~60,000 ph/MeV), which is used in medicine and high-energy physics [1]. The scintillation of GAGG:Ce is due to 5d–4f Ce^3+^ transitions, which give rise to emission band peaking at ~550 nm. Ce^3+^ decay time under 4f–5d(1) intracenter excitation is 40–60 ns [2,3]. Additional slow decay components usually arise at high-energy excitation which is related to delays at the thermalization and migration stages of energy transfer from the host to the activator. In particular, intermediate localization of charge carriers at shallow traps as well as energy transfer via Gd^3+^ states slow down the energy transfer processes [4,5].

Interaction of excitations is another effect which influences the scintillation response. When a scintillator absorbs high-energy particles, it converts their energy into electronic excitations, for which spatial distribution depends on the particles’ type and energy as well as the stopping power of scintillator. A high concentration of excitations in the local volume usually occurs at the end of the particles’ tracks and their interaction results in the non-proportional scintillation response to the energy of the ionizing particles and changes in scintillation decay kinetics [6,7]. The interaction between two excitations (e.g., excitons) can be described as an Auger-like dipole–dipole interaction, when one exciton annihilates while its energy is transferred to another one. The distance between excitons should be as small as the value of the radius of the dipole–dipole interaction (R_d-d_), which is of the order of few nanometers. 

In most cases, the non-exponential fast component(s) appears in the decay kinetics of compounds with intrinsic emission (e.g., self-trapped excitons (STEs) emission) while its intensity is suppressed due to the decrease in the number of excitations as a result of their interaction [8,9,10,11]. However, for CsI, it was shown that the luminescence intensity of 4.1 eV band increases quadratically with the increase the in excitation density which was connected with a new type of localized state of singlet exciton created through the “fusion” of self-trapped exciton and free exciton [12]. 

The influence of high-density excitation on the emission of Ce^3+^ was studied for CeF_3_ and Y_3_Al_5_O_12_ in [13,14,15]. It was shown that the interaction between excited Ce^3+^ ions, which are located in the neighboring sites of the CeF_3_ crystal lattice, results in the acceleration of decay kinetics in its initial part and in the suppression of the light yield, i.e., the behavior is similar to that observed in the case of the interaction of STEs. In contrast to CeF_3_, the high density excitation does not influence the Ce^3+^ decay time in the Y_3_Al_5_O_12_:Ce crystal as it contains cerium as an activator in concentrations much lower than those required for the interaction of neighboring Ce^3+^ ions. Non-linear dependence of Ce^3+^ luminescence intensity on excitation density has been detected for Y_3_Al_5_O_12_:Ce with the emission saturation at the excitation level of ~2.0 × 10^20^ eV/cm^3^ and it has been explained by mutual quenching of excitons created at high densities, preceding the stage of energy transfer to the Ce^3+^ ions. The influence of the high-density laser excitation of the activator emission has been also studied for CsI:Tl and NaI:Tl in [16]. Again, the decay time do not depend on the excitation density because the interaction occurs between STEs and only after interaction is the energy transferred to activator centers, which demonstrates luminescence with characteristic for Tl ions’ decay times. 

In contrast to Y_3_Al_5_O_12_:Ce and alkali halides, the presence of a gadolinium sublattice in gadolinium-containing crystals allows for the formation of additional pass-ways of energy transfer from the host to the emission centers. This will result in the appearance of delayed components in the emission decay, as was shown for cerium-doped gadolinium orthosilicates in [5]. One can expect redistributions between different pass-ways of energy transfer with the excitation density under interband excitation. Previously, the decay kinetics of GAGG:Ce were widely studied using high-energy sources such as X-ray, gamma, and electron beams and usually several decay components (corresponding to intracenter 5d–4f Ce^3+^ transitions as well as delayed ones) were detected [4,17,18,19,20,21]. However, in case of high energy excitation, a variety of excitations are created, whose energy and spatial distribution changes throughout the track of the high-energy particle (or quanta) absorbed in the GAGG:Ce. As a result, the distribution between different pass-ways to Ce^3+^ emission centers is averaged and the manifestation of density effects in decay kinetics is smeared.

The effect of the interaction of electronic excitations requires a high excitation density (>10^17^ cm^−3^) and can be studied using intense laser sources. Excitation with the 4th harmonic of a Ti:Saphire laser (6.2 eV) allows selective excitation in the energy region, which corresponds to the direct excitons’ creation in GAGG:Ce [18]. The selected energy as well as high excitation densities, which can be controlled and tuned up to 10^21^ cm^−3^, are distinctive properties of laser excitation sources, which allows for information on the excitations’ interaction and modification of energy transfer process with the excitation density to be obtained.

Here, we present a study of the interaction of electronic excitations in GAGG:Ce crystals under intense laser irradiation. The influence of high-density excitation on the decay characteristics of GAGG:Ce luminescence will be in the focus of the study.

## 2. Experimental Details

### 2.1. Crystal Growth

A single crystal with the composition in the melt Gd_2.97_Ce_0.03_Al_2_Ga_3_O_12_ (GAGG:Ce) was grown by the Czochralski method. The growth was performed in Ir crucibles in Ar atmosphere with 1–2 vol.% and additional amounts of O_2_. An optically polished crystal with a size of approximately 5 × 8 × 1 mm was cut perpendicular to the growth axis.

### 2.2. XRD Studies

Synchrotron XRD data for GAGG:Ce were measured in a large Debye−Scherrer camera at the BL15XU beamline of SPring-8 [22,23]. The intensity data were collected between 3.042° and 27.842° at 0.003° intervals in 2*Θ*; the incident beam was monochromatized at λ = 0.65298 Å. The sample was packed into a Lindemann glass capillary (inner diameter 0.1 mm), which was rotated during the measurement. The Rietveld analysis was performed using JANA2006 [24].

### 2.3. Luminescence Spectroscopy

The study of luminescence spectra and decay curves were performed at the CELIA laser center using a Ti:sapphire laser system, which generated 25 fs pulses at 800 nm with a frequency 1 of kHz. The luminescence was excited by the 4th harmonic (6.2 eV) of laser radiation. The collinear scheme was used for the harmonic generation. The filtering of the laser beam was achieved using the combination of dichroic mirrors and prisms. The density of created excitations in the laser spot center was controlled by the translation of the focusing lens and by the variation of laser beam energy. The lens with a 50 cm focal length was moved along the Z-axis which resulted in variation of the spot size (full width at half maximum) on the sample surface from 20 to 450 μm. The energy in the pulse was varied from 2 to 30 nJ in the 4th harmonic.

The peak density of electronic excitations was calculated using the following formula:

(1) $$N_{0}^{max}=1.34*10^{9}E(\frac{\alpha \sigma }{\pi a^{2}})$$

where E—beam energy (up to 30 nJ), *σ*—1 (number of excitations created by one photon), *α*—absorption coefficient, and *a*—beam waist. Measurement of the beam profile gives the 21 µm waist (full width at half maximum) of the beam with an M² better than 2 (see Figure S1 of Supporting Information).

The energy of the 4th harmonic—6.2 eV—corresponds to the fundamental absorption edge of GAGG:Ce. The value of absorption coefficient α was obtained from the Urbach fit of absorption spectrum previously performed for GAGG in [18]. The fit was performed for undoped GAGG because in a Ce-doped crystal, the fundamental absorption edge is distorted due to superposition with the Ce-related broad absorption band near the fundamental absorption edge. As a result, the α value at 6.2 eV was determined as 3000 cm^−1^ (see Figure S2 of Supporting Information). 

**Figure 1:**
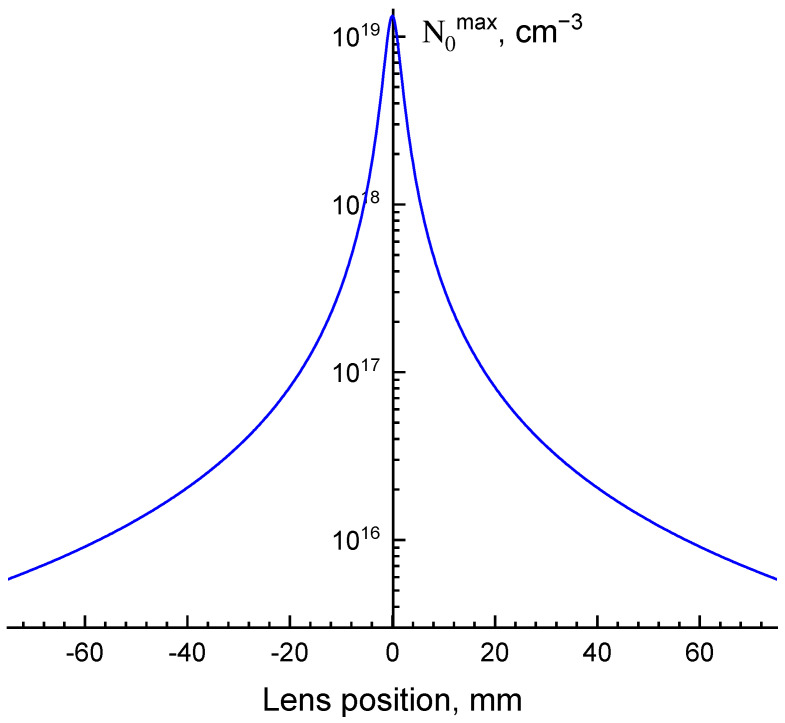
Dependence of maximal density of excitations on the translation of the focusing lens along the Z-axis of the laser beam, E = 20 nJ.

Considering all of the parameters, the variation in the density of excitations was determined using (1) as from ~10^15^ to ~10^19^ cm^−3^. The dependence of excitation density on the position of focusing lens is presented in Figure 1.

The samples were placed into a helium closed-cycle cryostat from ARS. All of the measurements were performed at 300 K. The luminescence was detected using the registration system, which consists of the TRIAX secondary monochromator from HORIBA Scientific (Kyoto, Japan) equipped with Hamamatsu MCP R3809U52 (Shizuoka, Japan) and intensified CCD camera Andor iStar (Belfast, UK). The luminescence spectra were not corrected on the registration sensitivity function.

The decay curves were measured using the MCP with 100 picosecond temporal resolution at the maximum of the Ce^3+^ emission band (525 nm). The luminescence spectra were measured using CCD in the whole temporal region 0–20,000 ns as well as in the time gating mode with time windows 0–50 ns (fast tw) and 200–5000 ns (slow tw), which were used to separate the influence of density effects on the “fast” emission which were characteristic for Ce^3+^ decay times and “slow” emission, which arise due to the delayed energy transfer to Ce^3+^. The measured dependences of Ce^3+^ emission intensity on the translation of the focusing lens along the Z-axis (Z-scans) are presented as intensity dependence on the excitation density using the data from Figure 1.

## 3. Results and Discussion

### 3.1. XRD Studies

It is well known that GAGG is a representative of the garnet-type structure (Ca_3_Al_2_(SiO_4_)_3_ [25]) described as A_3_B_2_(CO_4_)_3_. The structure is made up of *A*O_8_ polyhedra, *B*O_6_ octahedra, and *C*O_4_ tetrahedra. The Gd^3+^ cations occupy the *A*-positions of GAGG while the Ga^3+^ and Al^3+^ cations are located in the octahedral and tetrahedral sites. A synchrotron XRD study of GAGG:Ce was performed on the same sample as the one that was studied by powder XRD [18] to compare the structure refinement results from two datasets. Site occupation and the fractional atomic coordinates of GAGG:Ce from structure refinement using powder XRD data were used as an initial model. Occupancy of Ga^3+^ and Al^3+^ over the *B* and *C* positions was refined considering their multiplicities (*B* = *m*Ga^3+^ + (1 − *m*)Al^3+^, *C* = *n*Ga^3+^ + (1 − *n*)Al^3+^). As a result, the composition was determined to be Gd_3_Al_2.23_Ga_2.77_O_12_, which is slightly different from the powder XRD results (Gd_3_Al_2.3_Ga_2.7_O_12_).

**Figure 2:**
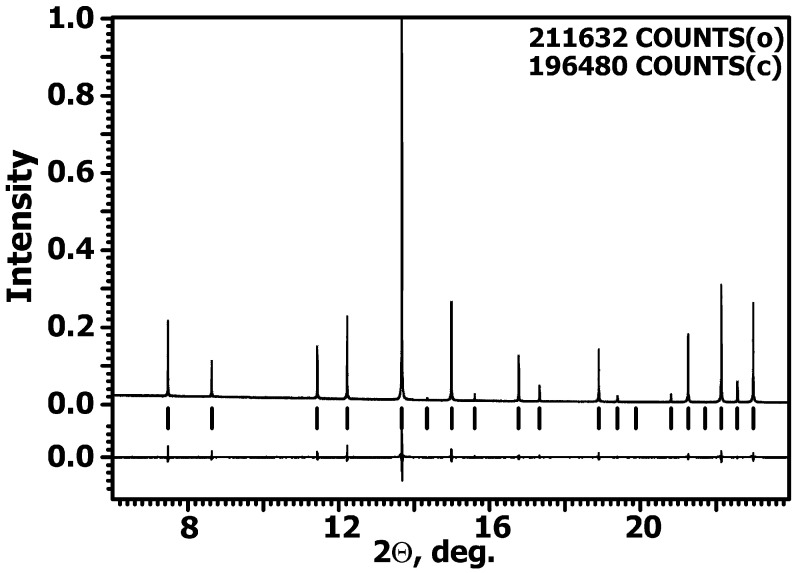
Fragments of the observed, calculated, and difference XRD patterns for Gd_3_Al_2.22_Ga_2.78_O_12_:Ce. Tick marks denote the peak positions of possible Bragg reflections.

The reliability factors (*R*-factors) show a good agreement between the calculated and the experimental XRD patterns. Figure 2 shows a portion of the observed, calculated, and difference XRD patterns for GAGG:Ce. The crystallographic data and refinement results for GAGG:Ce are presented in Table 1. The site occupation, fractional atomic coordinates, isotropic displacement atomic parameters (*U*_iso_), and anisotropic atomic displacement parameters for GAGG:Ce are listed in Table S1 of the Supporting Information. The main relevant interatomic distances are shown in Table S2 of the Supporting Information.

### 3.2. Influence of Excitation Density on the Decay Curves of GAGG:Ce

**Figure 3:**
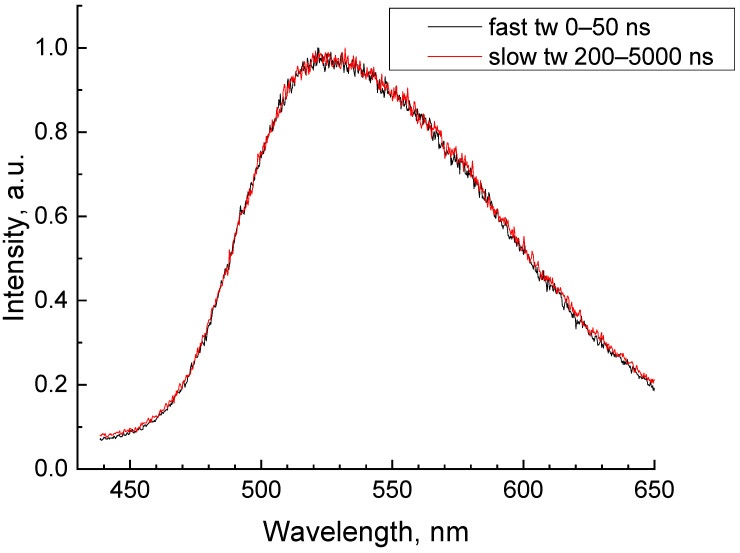
Luminescence spectra of GAGG:Ce measured at E_ex_ = 6.2 eV in fast (0–50 ns) and slow (200–5000 ns) time windows.

A broad non-elementary band peaking at 525 nm was observed in the luminescence spectra of GAGG:Ce (Figure 3). The band arose due to 5d(1)–4f transitions within Ce^3+^ ions. The band profile does not depend on the excitation density as well as on the time windows used for the experiment.

**Figure 4:**
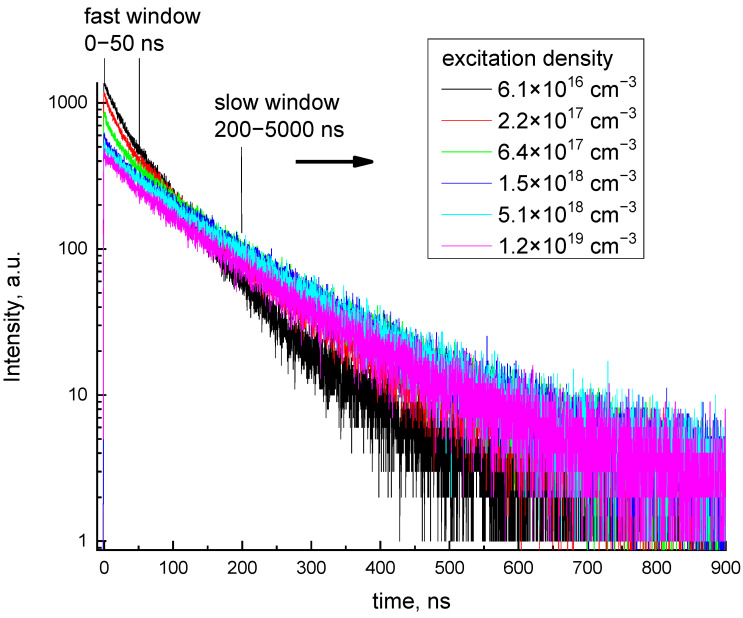
Decay curves of GAGG:Ce measured at the excitation density in the range from *N*_0_*^max^* = 3.6 × 10^16^ cm^−3^ to 7 × 10^18^ cm^−3^, E_ex_ = 6.2 eV.

**Figure 5:**
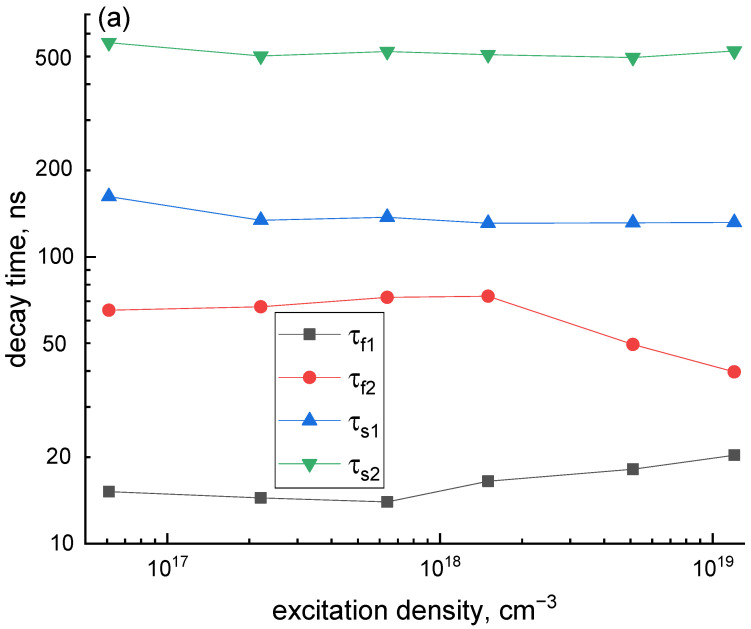

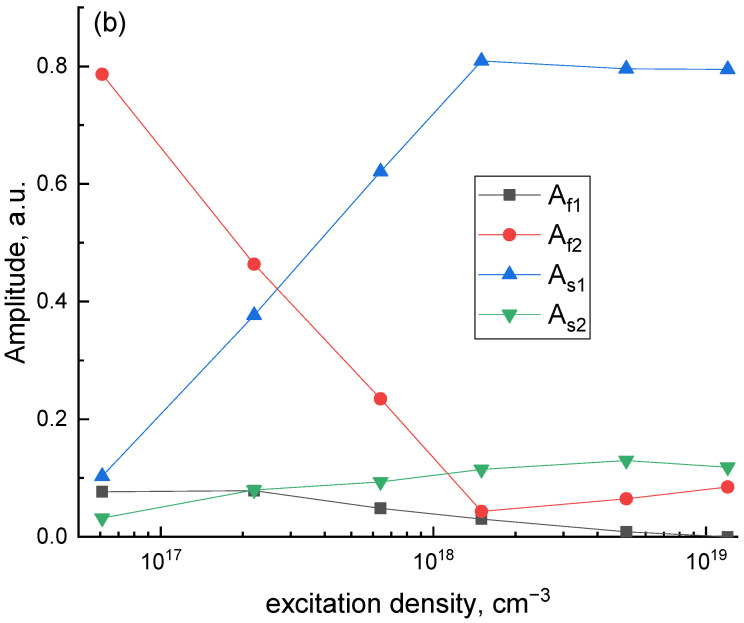
The dependence of the decay curves fitting parameters—decay times (**a**) and amplitudes (**b**) on the excitation density.

A set of decay curves obtained for the excitation densities from $N_{0}^{max}$ = 6.1 × 10^16^ cm^−3^ (lens out of focus) to $N_{0}^{max}$ = 1.2 × 10^19^ cm^−3^ (lens in focus) are presented in Figure 4. From the figure, the initial part of the decay curve is supressed while it increases for the τ > 200 ns with an increase in excitation density. The fit of the decay curves with the sum of four exponential components has been performed (Figure S3 of Supporting Information). The curves can be fitted by the same set of decay components, two fast (τ_f1_, τ_f2_) and two slow (τ_s1_, τ_s2_), regardless of the excitation density. The characteristic decay times were τ_f1_ = 16.4 ± 2.4, τ_f2_ = 61.1 ± 13.5, τ_s1_ = 138 ± 12, and τ_s2_ = 517 ± 22 ns, Figure 5a. The fastest component τ_f1_ is artificial and arises due to the electronic distortion of the decay curve, namely pulse miscounts during the dead time after pulse registration. Another fast decay component τ_f2_ ~ 60 ns corresponds to the characteristics of 5d–4f intracenter electronic transitions in Ce^3+^ ions. Two slower components (τ_s1_, τ_s2_) arise due to the delayed energy transfer from the host to Ce^3+^ ions. In contrast to the decay times, their amplitudes depend on the excitation density (Figure 5b). The amplitudes of two fast components decrease while those of slow ones increase with the increase in excitation density. 

It is worth noting that the increase in the excitation density does not result in the acceleration of the initial part of the decay curve as was reported for CeF_3_ [13]. This indicates that cerium ions are not directly involved in the interaction processes and the interaction occurs between excitons directly created under 6.2 eV laser excitation and the energy is transferred to Ce^3+^ only after the interaction. 

### 3.3. Z-Scans of GAGG:Ce

**Figure 6:**
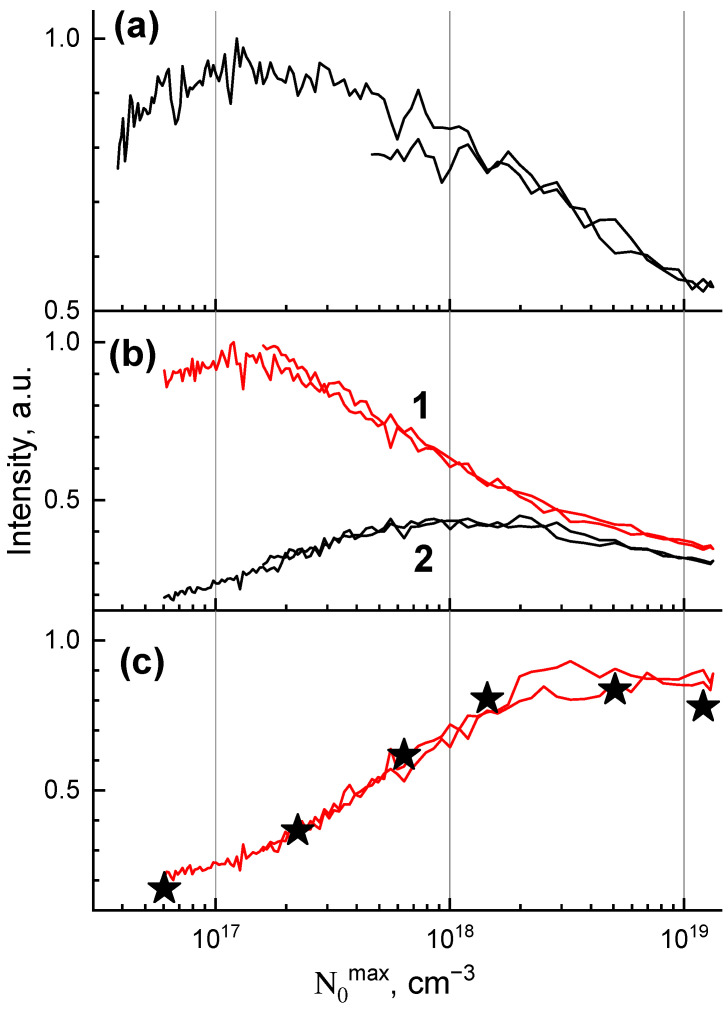
Dependence of GAGG:Ce emission intensity on the excitation density (Z-scan) measured in the whole temporal region (**a**) and in the fast (1) and slow (2) time windows (**b**). The curve (**c**) presents the ratio of dependences measured in slow and fast time windows. The ratio between the amplitudes of fast and slow components of the decay curve fit are given by asterisks.

The dependence of the Ce^3+^ emission intensity measured in the whole temporal region (0–20,000 ns) on the density of excitations (Z-scan) is presented in Figure 6a. The luminescence intensity decreases with the excitation density increase that is related to exciton–exciton interaction. The threshold excitation density needed for the luminescence suppression is only $N_{0}^{max}$ =2 × 10^17^ cm^−3^. Such a value implies a relatively low concentration of excitations. The mean distance between the excitations for this excitation can be estimated as $\frac{1}{\sqrt[{3}]{N_{0}^{max}}}$ ~17 nm. The calculated value exceeds the typical values of dipole–dipole interaction distances (R_d-d_ < 5 nm) by several times [8,9,10,11]. Therefore, we suppose that the interaction occurs between mobile excitons in the case of GAGG:Ce. 

Time-resolved Z-scans measured in the fast (0–50 ns) and slow (200–5000 ns) time windows are presented in Figure 6b. For the fast time window, the curve is similar to that obtained for the whole temporal region (Figure 6a). In contrast, for the slow time window, an increase in intensity is observed for the densities up to ~10^18^ cm^−3^. The effect is saturated at $N_{0}^{max}$ ~ 2 × 10^18^ cm^−3^ which can be deduced from the constant in the ratio between fast and slow time windows (Figure 6c). The ratio between delayed and prompt decay components was also determined from the fit of decay components presented in Figure 5 as (τ_s1_A_s1_ + τ_s2_A_s2_)/(τ_f1_A_f1_ + τ_f2_A_f2_). The corresponding data are presented by asterisks in Figure 6c and they coincide with the curve obtained from the time-resolved Z-scan experiment. 

### 3.4. Discussion

**Figure 7:**
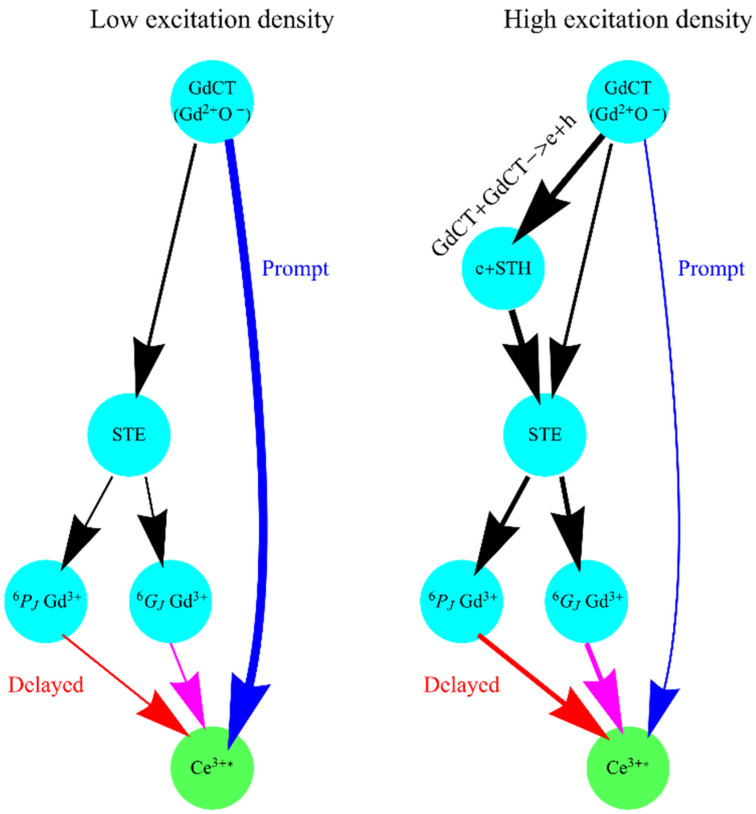
Scheme of the energy transfer pass-ways from the excitons to Ce^3+^ emission centers in GAGG:Ce in case of low (at **left**) and high (at **right**) excitation densities. GdCT—Gd charge transfer exciton; STE—self-trapped exciton; STH—self-trapped hole, Ce^3+^*-excited cerium ion.

According to the presented data, the decay kinetics of Ce^3+^ emission in GAGG:Ce depend on the excitation density. The decay becomes slower with the increase in the excitation density. No additional decay components arise according to the presented fit but the redistribution between the amplitudes of fast and delayed decay components takes place. This means that no additional channels of energy relaxation are created under excitation density increase but the redistribution between the existing energy channels occurs. There are several channels of the energy transfer from the host to Ce^3+^ emission centres in GAGG. The scheme of the energy transfer pass-ways is presented in Figure 7. The excitation energy 6.2 eV corresponds to the region of the direct exciton creation. The most probably Gd charge transfer (GdCT) excitons are created on the electron transition from 2p states of oxygen, which form the valence band top, to 5d states of Gd, which probably form the bottom of the conduction band similarly to Gd_3_Ga_5_O_12_ and Gd_3_Al_5_O_12_ [26]. Energy transfer from the GdCT exciton to cerium may occur in two ways: (1) directly from GdCT (it is a prompt excitation) and (2) via self-trapping of the GdCT exciton (STE) with further slow energy transfer to Ce^3+^ via 4f states of Gd^3+^. The former channel of energy transfer dominates under the excitation with a low density of laser irradiation. The low electron mass at the conduction band bottom in Gd_3_Ga_5_O_12_ and Gd_3_Al_5_O_12_ [26] implies high mobility of GdCT and low probability of their self-trapping.

The energy transfer via the second channel will result in the appearance of delayed decay component(s). As was shown in [5] for cerium-doped gadolinium oxyorthosilicate, there are two delayed components with decay times up to 194 and 736 ns, which arise due to the energy transfer via ^6^I_J_ and ^6^P_J_ states of gadolinium. Two delayed components with similar decay times—150 and 600 ns—were also obtained by the fit of experimental decays for GAGG:Ce. Their origin is ascribed to the energy transfer from the exciton to Ce^3+^ via ^6^I_J_ and ^6^P_J_ Gd^3+^ states as well. 

In the case of high excitation density, two mobile excitons may interact with the annihilation of the first one and disintegration of the second one into a separated electron and a hole. Such interaction takes place in GAGG:Ce which was proven by the Ce^3+^ intensity decrease in Z-scan experiments (Figure 6). The appearance of separated e-h pairs redistributes the contributions of the prompt and delayed energy transfer channels. We suppose that the hole can be self-trapped (formation of STH in Figure 7), as has previously been shown for garnets [27]. The electrons will be bound with STHs with the formation of self-trapped excitons. According to the model, self-trapped excitons transfer their energy via the Gd^3+^ subsystem, i.e., via delayed transfer. The number of excitations which are subjected to the process of exciton–exciton interaction quadratically increases with the excitation density while the number of excitations linearly grows with the density. This is a reason of redistribution between the amplitudes of slow and fast decay components with the increase in excitation density (Figure 5). As a result, the increased contribution of delayed components in the decay curves of Ce^3+^ emissions is obtained. The increase in the ratio is saturated at $N_{0}^{max}$ > 2 × 10^18^ cm^−3^ which is related to the reaching of a balance between the delayed and prompt energy transfer channels.

In order to demonstrate the described excitation density effect, we can write the simplified set of rate equations for concentrations of gadolinium charge transfer excitons $n_{\mathrm{GdCT}}\left(t\right)$, Gd ions with excitation at 4f subsystem $n_{\mathrm{Gd}4f}\left(t\right)$, STEs $n_{\mathrm{STE}}\left(t\right)$, and excited cerium ions $n_{\mathrm{Ce}}\left(t\right)$:

(2) $$\begin{array}{l} \frac{dn_{\mathrm{Ce}}\left(t\right)}{dt}=-\frac{1}{\tau_{\mathrm{Ce}}}n_{\mathrm{Ce}}\left(t\right)+\beta_{\mathrm{GdCT}\to \mathrm{Ce}}n_{Ce}^{0}n_{\mathrm{GdCT}}\left(t\right)+\beta_{\mathrm{Gd}4f\to \mathrm{Ce}}n_{Ce}^{0}n_{\mathrm{Gd}4f}\left(t\right),\\ \frac{dn_{\mathrm{GdCT}}\left(t\right)}{dt}=-\beta_{\mathrm{GdCT}\to \mathrm{Ce}}n_{Ce}^{0}n_{\mathrm{GdCT}}\left(t\right)-w_{\mathrm{GdCT}\to \mathrm{Gd}4f}n_{\mathrm{GdCT}}\left(t\right)-2\beta_{\mathrm{GdCT}+\mathrm{GdCT}\to \mathrm{STE}}n_{\mathrm{GdCT}}^{2}\left(t\right),\\ \frac{dn_{\mathrm{Gd}4f}\left(t\right)}{dt}=-\beta_{\mathrm{Gd}4f\to \mathrm{Ce}}n_{Ce}^{0}n_{\mathrm{Gd}4f}\left(t\right)-\frac{1}{\tau_{\mathrm{Gd}4f}}n_{\mathrm{Ce}}\left(t\right)+w_{\mathrm{GdCT}\to \mathrm{Gd}4f}n_{\mathrm{GdCT}}\left(t\right)+w_{\mathrm{STE}\to \mathrm{Gd}4f}n_{\mathrm{STE}}\left(t\right),\\ \frac{dn_{\mathrm{STE}}\left(t\right)}{dt}=-w_{\mathrm{STE}\to \mathrm{Gd}4f}n_{\mathrm{STE}}\left(t\right)+\beta_{\mathrm{GdCT}+\mathrm{GdCT}\to \mathrm{STE}}n_{\mathrm{GdCT}}^{2}\left(t\right) \end{array}$$

with initial conditions

$$n_{\mathrm{GdCT}}\left(0\right)=n_{0},\;n_{\mathrm{Gd}4f}\left(0\right)=0,\;n_{\mathrm{STE}}\left(0\right)=0,\;n_{\mathrm{Ce}}\left(0\right)=0.$$

**Figure 8:**
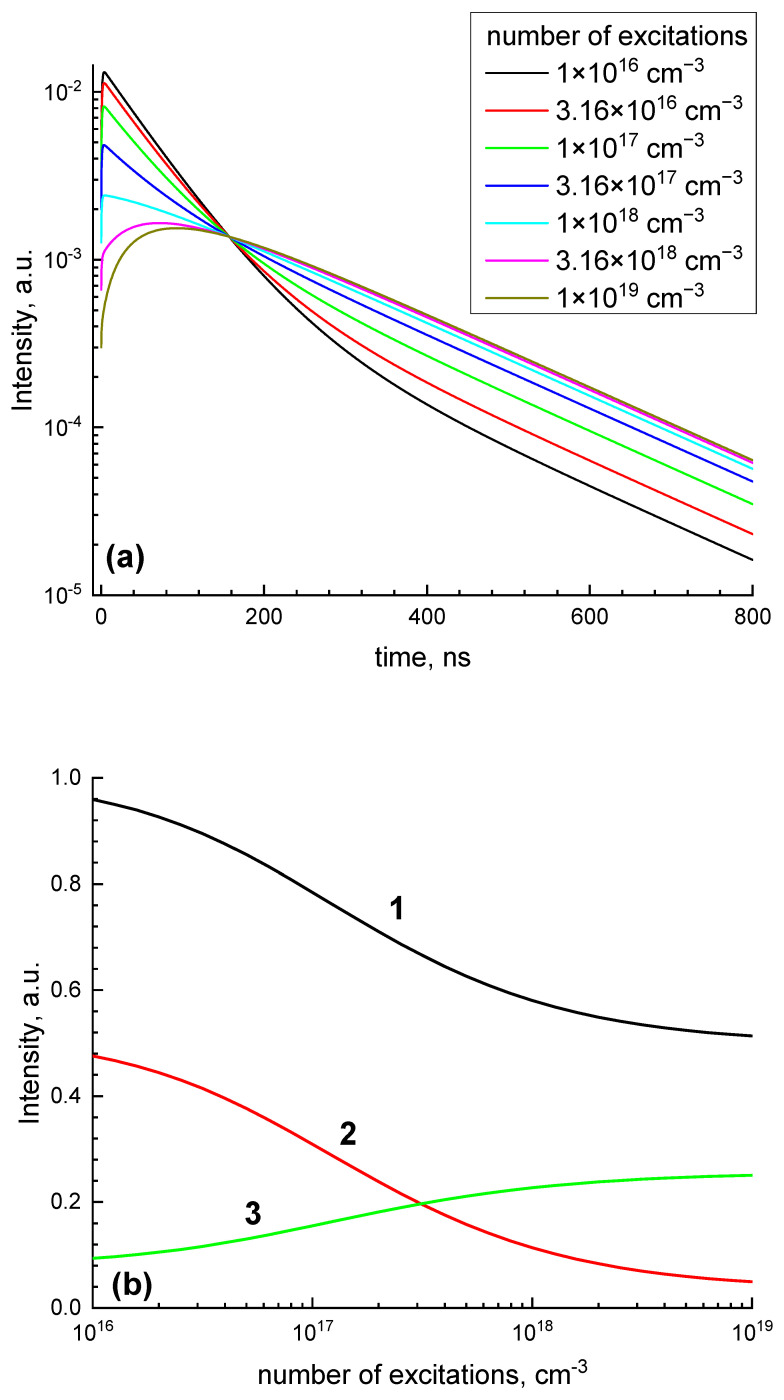
Results of numerical simulation using Equation (2) of the dependencies of decay kinetics (**a**) and emission intensity (**b**), total (1) and time-resolved in time windows 0–50 ns (2) and 200–5000 ns (3) on the initial concentrations of excitations.

Here, $\tau_{\mathrm{Ce}}$ and $\tau_{\mathrm{Gd}4f}$—radiation time of excited cerium and gadolinium, $\beta_{i\to j}$—bi-molecular rates of the process i→j, $n_{Ce}^{0}$—concentration of cerium in a non-excited state, $w_{i\to j}$—monomolecular rates of conversion of GdCT and STE into 4f excitation of gadolinium. Please note that in reaction GdCT + GdCT → STE, two excitations disappear and only one is created (pay attention to factor 2 in the last term of the second equation). This set of equations was solved for the set of parameters $\tau_{\mathrm{Ce}}$ = 60 ns, $\tau_{\mathrm{Gd}4f}$ = 100 μs, $\beta_{\mathrm{Gd}4f\to \mathrm{Ce}}n_{Ce}^{0}$ = 1/(200 ns), $\beta_{\mathrm{GdCT}\to \mathrm{Ce}}n_{Ce}^{0}$ = 1 ns^−1^, $w_{\mathrm{GdCT}\to \mathrm{Gd}4f}$ = 0.1 ns^−1^, $w_{\mathrm{STE}\to \mathrm{Gd}4f}$ = 1 ns^−1^, and $\beta_{\mathrm{GdCT}+\mathrm{GdCT}\to \mathrm{STE}}$ = 10^−17^ cm^3^/s. The results of the solutions for the initial concentration of excitations $n_{0}$ from 10^16^ to 10^19^ cm^−3^ are presented in Figure 8a (decay kinetics for different initial concentrations) and Figure 8b (dependence of total integral of cerium emissions and partial integrals for 0–50 ns and 200–5000 ns intervals). Decay curves are normalized to the initial concentration of excitations. The results of the numerical simulation qualitatively reproduce the experimental results thus justifying the proposed model of energy relaxation in GAGG:Ce.

## 4. Conclusions

The luminescence properties of GAGG:Ce single crystals were studied using the 4th harmonic of a Ti:saphire laser. It was shown that the emission intensity and decay characteristics of Ce^3+^ luminescence depend on the excitation density. The intensity of luminescence decreases with the excitation density that is related to exciton–exciton interaction. It is estimated that the exciton–exciton interaction starts when the average distance between excitations is 16 nm thus implying interaction between mobile (not self-trapped) excitons. The redistribution between the decay components of the Ce^3+^ emission occurs with the excitation density. In particular, the contribution of prompt components in the decay of the Ce^3+^ emission decreases and that of delayed components increases with the excitation density. The observed redistribution between decay components is connected with the increase in the probability of delayed energy transfer to Ce^3+^ via 4f levels of the Gd^3+^ sublattice when the interaction between excitations is realized. Numerical simulation of the modification of the energy transfer process with an excitation density has been performed, which qualitatively reproduces the experimental results and justifies the proposed model of energy relaxation.

## Figures and Tables

**Table 1:** Crystallographic data and refinement results for the Gd_3_Al_2.23_Ga_2.77_O_12_:Ce structure (SG *Ia*$\bar{3}$*d*, Z = 8).

Composition	Gd_3_Al_2.23_Ga_2.77_O_12_
Lattice parameters: *a*, Å	12.26005(1)
Unit cell volume, Å^3^	1842.794(3)
Calculated density, g/cm^3^	6.61(9)
R and R_w_ (%) for Bragg reflections (R_all_/R_obs_)	1.45/1.34 and 2.26/2.24
2*θ* range (^o^)	3.042–27.841
Step scan (2*θ*)	0.003
*I*_max_	211,632
№ reflec. (All/Obs.)	34/32
R_P_ and R_wP_; R_exp_ (%)	3.18, 4.93, 1.75
Goodness of fit (ChiQ)	2.82
Max./min. res. density (*e* × Å^−3^)	0.23/−0.26

## Data Availability

The data presented in this study are available on request from the corresponding author.

## Supplementary Materials

The following supporting information can be downloaded at: https://www.mdpi.com/article/10.3390/ma16030971/s1.

## References

[1] Lecoq P. (2016). Development of new scintillators for medical applications. Nucl. Instrum. Methods Phys. Res. Sect. A.

[2] Ogiegło J.M., Katelnikovas A., Zych A., Justel T., Meijerink A., Ronda C.R. (2013). Luminescence and Luminescence Quenching in Gd_3_(Ga,Al)_5_O_12_ Scintillators Doped with Ce^3+^. J. Phys. Chem. A.

[3] Bartosiewicz K., Babin V., Kamada K., Yoshikawa A., Kurosawa S., Beitlerova A., Kucerkova R., Nikl M., Zorenko Y. (2019). Ga for Al substitution effects on the garnet phase stability and luminescence properties of Gd_3_Ga_x_Al_5-x_O_12_:Ce single crystals. J. Lumin.

[4] Wu Y., Meng F., Li Q., Koschan M., Melcher C.L. (2014). Role of Ce^4+^ in the Scintillation Mechanism of Codoped Gd_3_Ga_3_Al_2_O_12_:Ce. Phys. Rev. Appl..

[5] Suzuki H., Tombrello T.A., Melcher C.L., Peterson C.A., Schweitzer J.S. (1994). The role of gadolinium in the scintillation processes of cerium-doped gadolinium oxyorthosilicate. Nucl. Instrum. Methods Phys. Res. Sect. A.

[6] Vasil’ev A.N., Gektin A.V. (2014). Multiscale Approach to Estimation of Scintillation Characteristics. IEEE Trans. Nucl. Sci..

[7] Bizarri G., Moses W.W., Singh J., Vasil’ev A.N., Williams R.T. (2009). The role of different linear and non-linear channels of relaxation in scintillator non-proportionality. J. Lumin..

[8] Kirm M., Nagirnyi V., Feldbach E., De Grazia M., Carre B., Merdji H., Guizard S., Geoffroy G., Gaudin J., Fedorov N. (2009). Exciton-exciton interactions in CdWO_4_ irradiated by intense femtosecond vacuum ultraviolet pulses. Phys. Rev. B.

[9] Laasner R., Fedorov N., Grigonis R., Guizard S., Kirm M., Makhov V., Markov S., Nagirnyi V., Sirutkaitis V., Vasil’ev A. (2013). Band tail absorption saturation in CdWO_4_ with 100 fs laser pulses. J. Phys. Condens. Matter.

[10] Markov S., Nagirnyi V., Vasil’ev A., Makhov V., Laasner R., Vielhauer S., Kirm M., Grigonis R., Sirutkaitis V. (2012). Modelling of decay kinetics of self-trapped exciton luminescence in CdWO_4_ under femtosecond laser excitation in absorption saturation conditions. Open Phys..

[11] Spassky D., Vasil’ev A., Belsky A., Fedorov N., Martin P., Markov S., Buzanov O., Kozlova N., Slegel V. (2019). Excitation density effects in luminescence properties of CaMoO_4_ and ZnMoO_4_. Opt. Mater..

[12] Belsky A., Fedorov N., Gridin S., Gektin A., Martin P., Spassky D., Vasil’ev A. (2019). Time-resolved luminescence z-scan of CsI using power femtosecond laser pulses. Radiat. Meas..

[13] Nikl M., Mares J.A., Dusek M., Lecoq P., Dafinei I., Auffray E., Pazzi G.P., Fabeni P., Jindra J., Skoda Z. (1995). Decay kinetics of Ce^3+^ ions under gamma and KrF excimer laser excitation in CeF_3_ single crystals. J. Phys. Condens. Matter.

[14] Belsky A.N., Glukhov R.A., Kamenskikh I.A., Martin P., Mikhailin V.V., Munro I.H., Pedrini C., Shaw D.A., Shpinkov I.N., Vasil’ev A.N. (1996). Luminescence quenching as a probe for the local density of electronic excitations in insulators. J. Electron Spectrosc. Relat. Phenom..

[15] Krzywinski J., Andrejczuk A., Bionta R.M., Burian T., Chalupský J., Jurek M., Kirm M., Nagirnyi V., Sobierajski R., Tiedtke K. (2017). Saturation of a Ce:Y_3_Al_5_O_12_ scintillator response to ultra-short pulses of extreme ultraviolet soft X-ray and X-ray laser radiation. Opt. Mater. Express.

[16] Williams R.T., Grim J.Q., Li Q., Ucer K.B., Moses W.W. (2011). Excitation density, diffusion—Drift, and proportionality in scintillators. Phys. Status Solidi B.

[17] Kamada K., Yanagida T., Endo T., Tsutumi K., Usuki Y., Nikl M., Fujimoto Y., Fukabori A., Yoshikawa A. (2012). 2 inch diameter single crystal growth and scintillation properties of Ce:Gd_3_Al_2_Ga_3_O_12_. J. Cryst. Growth.

[18] Spassky D., Kozlova N., Zabelina E., Kasimova V., Krutyak N., Ukhanova A., Morozov V.A., Morozov A.V., Buzanov O., Chernenko K. (2020). Influence of Sc cation substituent on structural properties and energy transfer processes in GAGG:Ce crystals. CrystEngComm.

[19] Sibczynski P., Iwanowska-Hanke J., Moszyński M., Swiderski L., Szawłowski M., Grodzicka M., Szczęśniak T., Kamada K., Yoshikawa A. (2015). Characterization of GAGG:Ce scintillators with various Al-to-Ga ratio. Nucl. Instrum. Methods Phys. Res. Sect. A.

[20] Tamulaitis G., Vasil’ev A., Korzhik M., Mazzi A., Gola A., Nargelas S., Vaitkevičius A., Fedorov A., Kozlov D. (2019). Improvement of the Time Resolution of Radiation Detectors Based on Gd_3_Al_2_Ga_3_O_12_ Scintillators with SiPM Readout. IEEE Trans. Nucl. Sci..

[21] Prusa P., Kucera M., Mares J.A., Onderisinova Z., Hanus M., Babin V., Beitlerova A., Nikl M. (2015). Composition Tailoring in Ce-Doped Multicomponent Garnet Epitaxial Film Scintillators. Cryst. Growth Des..

[22] Tanaka M., Katsuya Y., Yamamoto A. (2008). A new large radius imaging plate camera for high-resolution and high-throughput synchrotron X-ray powder diffraction by multiexposure method. Rev. Sci. Instrum..

[23] Tanaka M., Katsuya Y., Matsushita Y., Sakata O. (2013). Development of a synchrotron powder diffractometer with a one-dimensional X-ray detector for analysis of advanced materials. J. Ceram. Soc. Jpn..

[24] Petricek V., Dusek M., Palatinus L. (2014). Crystallographic Computing System JANA2006: General features. Z. Krist.-Cryst. Mater..

[25] Abrahams S.C., Geller S. (1958). Refinement of the structure of a grossularite garnet. Acta Crystallogr..

[26] AFLOW. https://aflow.org/material/?id=aflow:0d48f6442e93b38d.

[27] Laguta V., Buryi M., Pejchal J., Babin V., Nikl M. (2018). Hole Self-Trapping in Y_3_Al_5_O_12_ and Lu_3_Al_5_O_12_ Garnet Crystals. Phys. Rev. Appl..

