# Supplementary material for: Decay Kinetics of Gd3Al2Ga3O12:Ce3+ Luminescence under Dense Laser Irradiation
Source: Materials (Basel). 2023 Jan 20;16(3):971. doi: 10.3390/ma16030971 (PMC9917819; doi:10.3390/ma16030971)
Supplement: Supplementary file 1 [file materials-16-00971-s001.zip › materials-2108070-supplementary.pdf]

Supporting information for the manuscript

Decay kinetics of $Gd_3Al_2Ga_3O_{12}:Ce^{3+}$ luminescence under dense laser irradiation

Dmitry Spassky, Andrey Vasil'ev, Nataliya Krutyak, Oleg Buzanov, Vladimir Morozov, Alexei Belik, Nikita Fedorov, Patrick Martin and Andrey Belsky

Table S1. Site occupation, fractional atomic coordinates, isotropic (U_{iso}) and anisotropic atomic displacement parameters in $Gd_3Al_{2.23}Ga_{2.77}O_{12}$.

	**Atom**	**Occupancy**	***x***	***y***	***z***	***U*iso**
GAGG	*A*	Gd^{3+}	0.125	0	0.25	0.0063(4)
	B	0.399(7)Ga^{3+}+ 0.601(7)Al^{3+}	0	0	0	0.0068(15)
	C	0.658(8)Ga^{3+}+ 0.342(8)Al^{3+}	0.375	0	0.25	0.0033(13)
	O	O	0.2787(3)	-0.1006(3)	0.3001(3)	0.0000(16)
	U^{11}	U^{22}	U^{33}	U^{12}	U^{13}	U^{23}
Gd1	0.0050(9)	0.0069(6)	0.0069(6)	0	0	0.0022(8)

Table S2. Selected distances (Å) in $Gd_3Al_{2.23}Ga_{2.77}O_{12}$.

**Polyhedron**	**Distance, Å**	**GAGG**
AO_8	*A*-O×4	2.341(4)
	A-O×4	2.493(4)
BO_6	*B*-O×6	1.962(4)
CO_4	*C*-O×4	1.814(4)

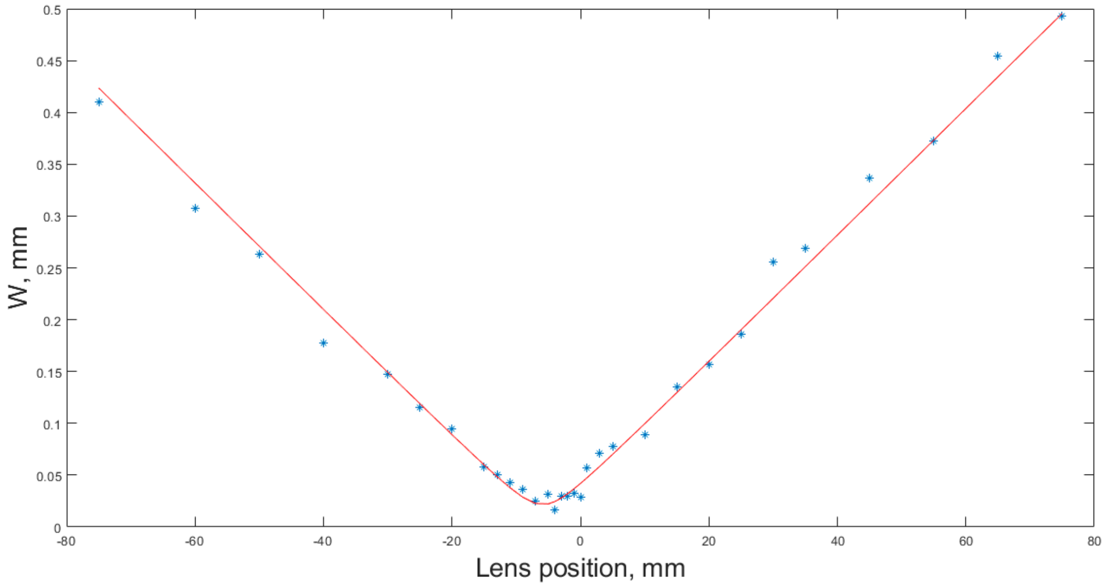

Figure S1. Dependence of the laser beam waist (FWHM) on the translation of the focusing lens along the z – axis.

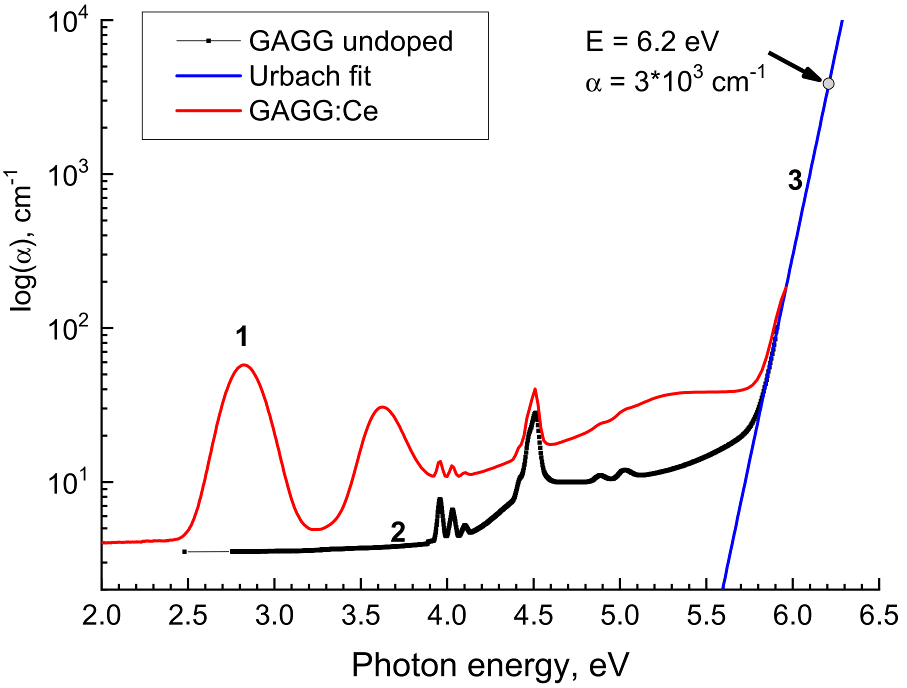

Figure S2. Absorption spectra of GAGG:Ce (1) and undoped GAGG (2) measured at 300 K. Urbach fit of the fundamental absorption edge of undoped GAGG crystal is presented by curve (3).

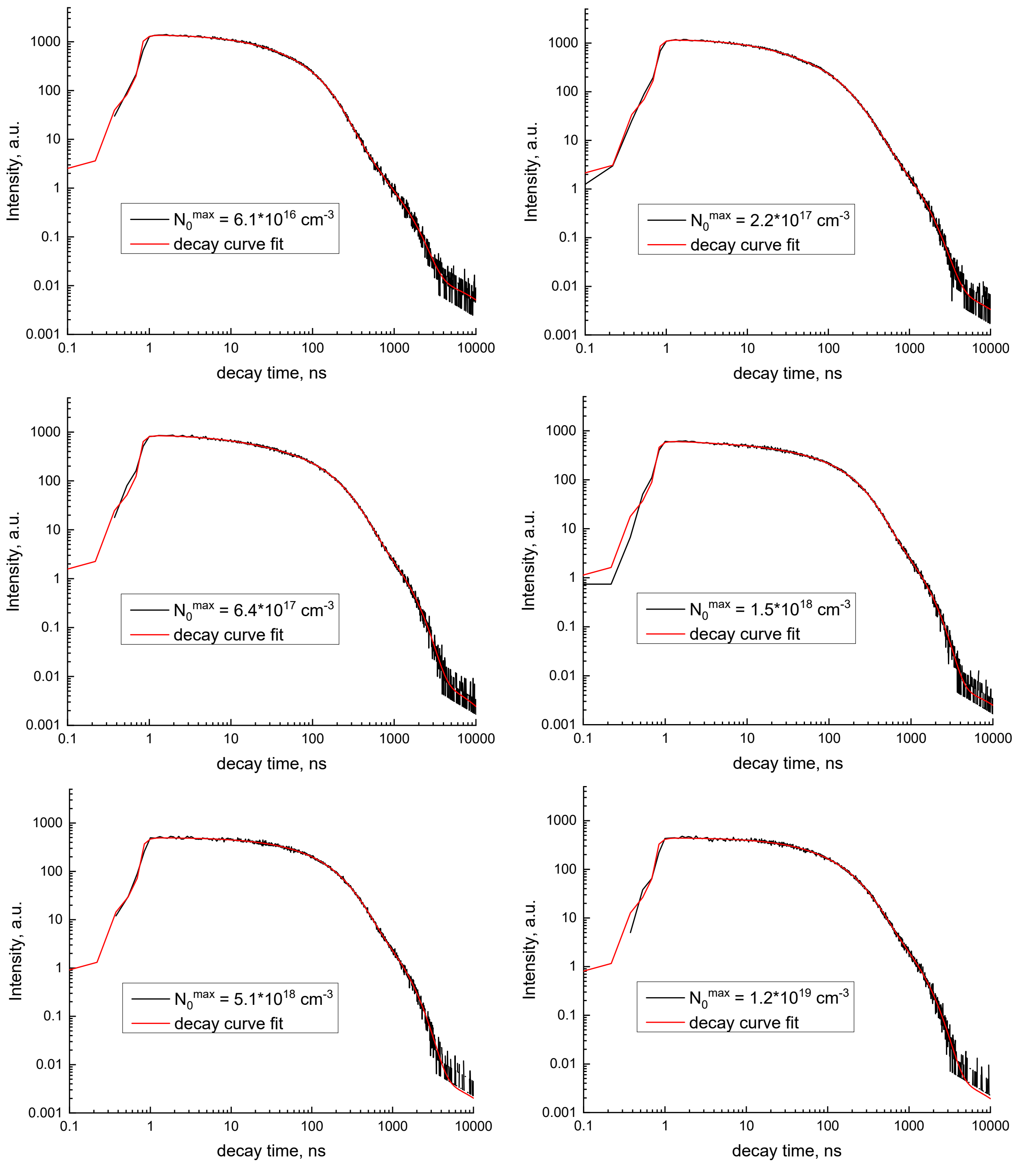

Figure S3. Decay curves (averaged, black curve) and their fit with the sum of four exponential curves with the parameters, presented in Figure 5 (red curve).
